# The Impact of 3′UTR Variants on Differential Expression of Candidate Cancer Susceptibility Genes

**DOI:** 10.1371/journal.pone.0058609

**Published:** 2013-03-05

**Authors:** Laura E. Skeeles, Jessica L. Fleming, Kimberly L. Mahler, Amanda Ewart Toland

**Affiliations:** 1 Department of Molecular Virology, Immunology and Medical Genetics, Comprehensive Cancer Center, The Ohio State University, Columbus, Ohio, United States of America; 2 Division of Human Genetics, Department of Internal Medicine, The Ohio State University, Columbus, Ohio, United States of America; Keio University, Japan

## Abstract

Variants in regulatory regions are predicted to play an important role in disease susceptibility of common diseases. Polymorphisms mapping to microRNA (miRNA) binding sites have been shown to disrupt the ability of miRNAs to target genes resulting in differential mRNA and protein expression. *Skin tumor susceptibility 5* (*Skts5*) was identified as a locus conferring susceptibility to chemically-induced skin cancer in NIH/Ola by SPRET/Outbred F1 backcrosses. To determine if polymorphisms between the strains which mapped to putative miRNA binding sites in the 3′ untranslated region (3′UTR) of genes at *Skts5* influenced expression, we conducted a systematic evaluation of 3′UTRs of candidate genes across this locus. Nine genes had polymorphisms in their 3′UTRs which fit the linkage data and eight of these contained polymorphisms suspected to interfere with or introduce miRNA binding. 3′UTRs of six genes, *Bcap29*, *Dgkb, Hbp1, Pik3cg, Twistnb*, and *Tspan13* differentially affected luciferase expression, but did not appear to be differentially regulated by the evaluated miRNAs predicted to bind to only one of the two isoforms. 3′UTRs from four additional genes chosen from the locus that fit less stringent criteria were evaluated. *Ifrd1* and *Etv1* showed differences and contained polymorphisms predicted to disrupt or create miRNA binding sites but showed no difference in regulation by the miRNAs tested. In summary, multiple 3′UTRs with putative functional variants between susceptible and resistant strains of mice influenced differential expression independent of predicted miRNA binding.

## Introduction

*Mus spretus* mice are resistant to skin cancer compared to *Mus musculus* mice. In previous linkage studies of dimethylbenz [a] anthracene (DMBA)/12-O-tetradecanoylphorbol-13-acetate (TPA)- induced skin cancer a number of skin cancer susceptibility loci were identified in SPRET/Outbred by NIH/Ola F1 backcross mice [1]–[4]. Linkage studies were also performed using SPRET/EiJ by FVB/N, SPRET/EiJ by NIH/Ola and STF/Pas by NIH/Ola F1 backcross mice which identified additional susceptibility loci. One of the skin susceptibility loci, *Skin tumor susceptibility 5* (*Skts5*), was found in the SPRET/Outbred by NIH/Ola crosses, but not in STF/Pas by NIH/Ola F1 or SPRET/EiJ by FVB/N crosses [3], [4]. Linkage results for SPRET/EiJ by NIH/Ola were equivocal for this region. Sequence analyses of 54 genes and coding elements across a 14-Mb peak linkage region at *Skts5* led to the identification of a number of coding changes consistent with the linkage analyses (Mahler et al. 2008).

Differential gene expression is postulated to be as important, if not more important, for disease susceptibility as non-synonymous coding changes [5]. Gene expression differences due to inherited factors may be caused by variations in enhancer or promoter binding sites, variations in epigenetic regulation impacting methylation or chromatin modifications, variations in expression of trans-acting factors or differences in regulation by microRNAs (miRNAs) [6]–[9]. Single nucleotide polymorphisms (SNPs) falling specifically in the 3′untranslated region (3′UTR) of genes may interfere with mRNA stability and translation through effects on polyadenylation and regulatory protein-mRNA and miRNA-mRNA interactions [10]–[12]. Preliminary studies in humans have identified variations in the 3′UTR of genes that appear to affect cancer risk by disrupting normal miRNA binding [13], [14]. One such variant in the *KRAS2* gene increases the risk for lung and ovarian cancer by changing the ability of miRNA *let-7* to bind [15], [16]. Another study in mice looked at miRNA complementary sites for three miRNAs that were introduced or disrupted by SNPs by allelic-imbalance sequencing [17]. Differences in expression fitting with the RNA-sequence data were observed for a large percentage of putative target genes, suggesting that variations in 3′UTRs have a key role in gene expression differences between individuals.

To determine if polymorphisms in putative miRNA binding sites were affecting gene expression and could be functional candidates for *Skts5*, we performed a systematic analysis of 3′UTR regions for the genes across the locus. We identified sequence variants in nine genes which fit with the linkage analysis (were polymorphic between SPRET/Outbred and NIH/Ola and not different between STF/PAS and NIH/Ola or SPRET/EiJ and FVB/N). SNPs from eight of these 3′UTRs were predicted to map to putative microRNA binding sites. An additional 18 genes were polymorphic between SPRET/Outbred and NIH/Ola and SPRET/EiJ and FVB/N but not between STF/PAS and NIH/Ola. Here, we describe the effects of variants from these candidate susceptibility genes on expression.

## Materials and Methods

### Animal material and cell lines

No living animals were used in this study; existing DNA and tissues were provided by collaborators or through commercial sources. The UCSF and University of Roswell Park Institutional Animal Care and Use Committees (IACUC) approved the original animal work which produced the samples used in this study. Laboratory origins of each of the strains of mice in the study are as follows: STF/PAS (inbred line maintained by the Institute Pasteur), SPRET/EiJ (inbred line maintained by the Jackson Laboratories, Bar Harbor, ME), outbred *spretus* (SPRET/Outbred, outbred line maintained by Hiroki Nagase, Roswell Park Institute), FVB/N (inbred line maintained by the Jackson laboratories) and NIH/Ola (inbred line maintained by Allan Balmain). NIH/Ola and SPRET/Outbred are homozygous across *Skts5*. Tissues for NIH/Ola mice were provided by Dr. Allan Balmain and tissues for SPRET/Outbred mice were provided by Hiroki Nagase. DNA was isolated from tails or spleens of SPRET/Outbred and NIH/Ola mice using standard methods [18]. SPRET/EiJ and FVB/N DNA and tissues were purchased from the Jackson Laboratories. STF/PAS DNA was a gift from Xavier Montagutelli, DVM, PhD of the Institut Pasteur. C5N, a non-immunogenic murine keratinocyte cell line obtained from Allan Balmain, was maintained in 1x Dulbecco's Modified Eagle Medium (DMEM) with 10% Fetal Bovine Serum (FBS) and 1% penicillin/streptomycin antibiotic.

### Sequence analysis

3′UTRs of the 39 genes mapping to *Skts5* for which we did not have sequence data for all strains used in the linkage analyses were identified using the Ensembl database, builds 35–48. We designed PCR primers using Integrated DNA Technology's SciTools PrimerQuest web-based program [http://www.idtdna.com/Scitools/Applications/Primerquest; San Diego, CA]. PCR products were treated with Exo/SAP-IT (USB, Cleveland, OH) to remove single stranded DNA. Automated sequencing of PCR products was conducted on an ABI 3700 (Applied Biosystems/Life Technologies, Carlsbad, CA) by standard methods. Primers used for PCR were also used for the sequencing. Forward and reverse sequence reads were analyzed and compared using Lasergene/DNAstar 3.0 (DNASTAR, Madison, WI). The sequence traces were inspected visually whenever a nucleotide substitution was indicated.

### Identification of potential microRNA binding sites

3′UTR polymorphisms that were observed only between NIH/Ola and SPRET/Outbred were evaluated to determine if they disrupted or introduced *in silico* predicted microRNA binding sites. Four programs were used: MiRanda (www.microRNA.org) [19], MicroInspector (http://bioinfo.uni-plovdiv.bg/microinspector/) [20], Patrocles finder (www.patrocles.org) [21] and MicroSNiPer (http://cbdb.nimh.nih.gov/microsniper/getSeqByNM.php) [22]. MiRanda allowed us to predict if our SNPs of interest disrupted miRNA binding sites in the mouse reference strain, which was highly similar to NIH/Ola 3′UTRs. The other three programs allow unique sequences to be analyzed for microRNA binding sites. For sites predicted by MicroInspector, we first considered those which had a predicted minimum free energy (MFE) of greater than −15 kcal/joule in one form and a MFE of less than −20 kcal/joule in the other form. When reevaluated, only those which were estimated to have a MFE of greater than −18 kcal/joule in one form and a MFE of −22 kcal/joule or less in the other form, as has been used previously for *Mus musculus* [20], were further tested experimentally. −22 kcal/J or less was considered strong binding and −18 kcal/J or higher was considered no or very weak binding. Patrocles and MicroSNiPer allow the comparison of two unique sequences for differences in predicted binding sites. MicroSNiPer requires SNPs to be entered into the prediction program, so this tool was unable to predict sites created or disrupted by insertions or deletions. Using MicroInspector, Patrocles, and MicroSNiPer, we picked candidate miRNAs that were predicted to bind to only the mouse strain (NIH/Ola or SPRET/Outbred), that demonstrated lower expression as measured by our 3′UTR luciferase assay expression and that contained a polymorphism in the miRNA binding site that fit with the mouse linkage results.

### Cloning

A section of each candidate gene 3′UTR which included polymorphic sites that fit with the mouse linkage and were predicted to interfere with miRNA binding was cloned into the Clontech pGL3 control vector (Mountain View, CA) (Table S1). The vector was linearized by inverse PCR (iPCR) using Advantage HD Polymerase Mix (Clontech), according to manufacturer's protocol. iPCR primers were designed using IDT PrimerQuest software. Linear products were separated from circular vector using the QIAquick Gel Extraction Kit (Qiagen, Frederick, MD). PCR primers for cloning using Clontech's In-fusion HD cloning kit protocol were designed using IDT PrimerQuest software (Table S2). PCR products were amplified from NIH/Ola and SPRET/Outbred DNA, purified, and cloned into the vector 3′ of the luciferase gene by recombination using Clontech's In-Fusion HD cloning kit, according to manufacturer's protocol. Plasmid DNA was analyzed by restriction digestion and clones were sequence verified by Sanger sequencing.

### Site-Directed Mutagenesis

For genes in which the NIH/Ola or SPRET/Outbred 3′UTR was difficult to clone, polymorphic sites which fit with the mouse linkage were changed by site-directed mutagenesis (SDM) using plasmids containing the 3′UTR of the other strain as template. SDM primers were designed using Stratagene's QuikChange Primer Design Program, and SDM was performed using Strategene's QuikChange Lightning Multi Site-Directed Mutagenesis Kit per manufacturer's recommended conditions (Agilent, Santa Clara, CA). Mutated plasmids were sequence verified.

### Transfections/luciferase assays

C5N cells were co-transfected with the pGL3 In-Fusion products, pRL-TK Renilla firefly luciferase vector (Promega, Madison, WI), and pre-miRNAs or MirVana mimic miRNA precursors (Ambion/Life Technologies, Grand Island, NY) for miRNA assays. Transfections were performed using Lipofectamine with Plus Reagent (Invitrogen/Life Technologies, Grand Island NY) for 3′UTR DNA transfections and Lipofectamine 2000 (Invitrogen) for transfections using plasmids (600 ng/well of each plasmid) and miRNA precursors or scrambled controls (13 pmole/well for a 12-well plate). Twenty-four hours post-transfection, protein was isolated using M-PER (Thermo Scientific/Pierce, Rockford, IL), and RNA was isolated using Ribozol (ISC Bioexpress, Kaysville, UT). For luciferase measurements, a D-Luciferin (Sigma-Aldrich, Saint Louis, MO) mix, containing DTT and glycylglycine was added to 30 µl isolated protein in a Vertias Microplate Luminometer using the Veritas Luciferase Assay program (Promega, Madison, WI). To activate the reaction an ATP mix, with DTT, glycylglycine, EGTA, MgSO_4_, and K2PO_4_, was added. A control reporter assay included native Coelenterizine (NanoLight Technologies, Pinetop, AZ) with ATP, DTT, glycylglycine, EGTA, MgSO_4_, and K2PO_4_, added to 30 µl isolated protein following the Promega Renilla protocol. Luciferase relative light unit reads were normalized to Renilla luciferase. Luciferase ratios relative to mock-transfected cells are shown. Transfections and luciferase assays were performed a minimum of two times for each 3′UTR set of constructs and miRNA assay with the exception of *miR-3064-3p* (*Pik3cg*) which was only tested once and showed convincing results of no differences. Student t-tests were used to calculate significance of expression differences.

Additional experimental conditions were tested for transfections with the *Twistnb* 3′UTRs together with *miR-3074-5p* and/or *miR-691*. Experiments were performed as described above except that C5N cells were harvested at 24, 48, and 72 hours using a higher concentration of miRNA (26 pmoles) or both miRNAs together (26 pmoles each). A lower dose of transfected miRNA (7 pmoles) or both miRNAs together (7 pmole each) was also evaluated for an effect of *Twistnb* 3′UTRs at 24 and 48 hours. Additional experiments to assess the effects of *miR-3074-5p* on *Twistnb* isoforms included transfections in C5N cells with an anti-miRNA for *miR-3074-5p* or a negative control inhibitor at 24 and 48 hours posttransfection at two concentrations (13 pmoles and 30 pmoles).

### Confirmation of miRNA transfection by quantitative PCR (qPCR)

To confirm miRNA transfection, RNA was isolated as described above. Reverse transcription (RT) was performed using Applied Biosystem's Multiscribe RT kit according to manufacturer's recommended protocol (LifeTechnologies/Applied Biosystems, Carlsbad, CA). Taqman qPCR probes for miRNAs were purchased from Applied Biosystems. *Sno202* was used to calculate relative expression. qPCR was performed in triplicate for each sample. Experiments included no-RT and no template controls. Cycle threshold (CT) values were averaged across triplicates and delta CT values were calculated between each test miRNA and mock control.

### Identification of microRNA candidates

To refine our list of candidate microRNAs (miRNAs), we analyzed both the NIH/Ola and SPRET/Outbred forms of these binding sites in two minimum free energy (MFE) prediction tools, RNAhybrid (http://bibiserv.techfak.uni-bielefeld.de/rnahybrid/) [23], and RNAcofold (http://rna.tbi.univie.ac.at/cgi-bin/RNAcofold.cgi) [24]. We further refined our list of candidate 3′UTRs to those whose SNPs were predicted to induce a 5 kcal/J or greater difference in miRNAbinding MFE between NIH/Ola and SPRET/Outbred by both prediction tools (Table S3).

### Evaluation of mRNA expression by qPCR

To identify genes showing differential expression, RNA was isolated from tails of NIH/Ola and SPRET/Outbred mice using Trizol (Invitrogen) according to manufacturer's recommended conditions. One microgram of RNA from each animal was reversed transcribed using iScript cDNA synthesis kit (Bio-Rad, Hercules, CA). To assess mRNA expression of candidate *Skts5* genes, Taqman probes were purchased from Applied Biosystems (Life Sciences Technologies). Each sample was measured in triplicate. Three control genes, *L19*, *Ppia* and *Hprt*, were used to calculate relative expression and the average difference in expression was calculated across all three controls. Experiments included water blanks and no-RT controls. Significance of differential expression was determined by Student T-tests.

## Results

### Identification of candidate 3′UTRs for study

*Skts5* is a skin tumor susceptibility locus previously identified in F1 backcross mice between susceptible NIH/Ola and SPRET/Outbred strains [4], [25]. Linkage to this locus was not found in F1 backcrosses between NIH/Ola and STF/PAS or between FVB/N and SPRET/EiJ, other skin resistant strains of Mus spretus suggesting that potentially functional sequence variants which were present in SPRET/Outbred, but not in STF/PAS or SPRET/EiJ, could be considered as candidate variants. In previous studies, we sequenced coding exons for 54 genes at the minimal *Skts5* linkage region in strains of mice susceptible to skin cancer (NIH/Ola and FVB/N) and mice resistant to skin cancer (SPRET/Outbred, SPRET/EiJ, STF/PAS) [4]. In our initial study, we did not complete genotyping of the entire 3′UTRs for all genes in all of the strains of mice used in the study. To generate missing sequence data, we sequenced the longest 3′UTR version of 39 genes for the strains for which we were missing data, primarily STF/PAS. Of the genes assessed, there were 24 3′UTR polymorphisms from nine genes which fit the most conservative linkage data in that they were only polymorphic between NIH/Ola and SPRET/Outbred, but were not polymorphic between STF/PAS and NIH/Ola or between FVB/N and SPRET/EiJ (Table 1).

**Table 1:** 3′UTR SNPs only in SPRET/Outbred.

Gene	Position	Change	RS#	NIH/Ola	FVB/N	SPRET/O	SPRET/EIJ	STF/PAS
*Bcap29*	1409	C>T		C	C	T	C	C
	1423	G>A		G	G	A	G	G
*Cbll1*	1758	Ins A	rs108265667	-	-	InsA	-	-
*Dgkb*	3117	A>T		A	A	T	A	A
	3170	T>G		T	T	G	T	T
	4502	C>G		C	C	G	C	C
	5181	7bp del		-	-	Del 7	-	-
*Hbp1*	2437	G>C		G	G	C	G	G
	2626	7bp del		-	-	Del 7	-	-
*Meox2*	1756	Ins T	*rs107810376*	-	-	Ins T	-	-
	2032	T>C	*rs108906552*	T	T	C	T	T
*Pik3cg*	4978	C>T		C	C	T	C	C
	4979	C>G		C	C	G	C	C
	5841	3bp del		-	-	Del 3	-	-
*Stxbp6*	1012	40bp del		-	-	Del 40	-	-
	1078	G>T		G	G	T	G	G
	1198	20bp del		-	-	Del 20	-	-
	1329	G>A		G	G	A	G	G
	1875	A>C		A	A	C	A	A
	2236	C>T		C	C	T	C	C
	2344	Del AG		-	-	Del AG	-	-
*Tspan13*	958	Ins/Del		Ins T	Ins T	Ins TTT	-	Ins TT
	1207	A>C		A	A	G	A	A
*Twistnb*	1489	A>G	*rs107692237*	A	A	G	A	A

Position, nucleotide position in gene according to the Ensembl database, SPRET/O, SPRET/Outbred; -, no insertion or deletion; Ins, insertion; Del, deletion.

### Effect of 3′UTR variants on expression

We expected that only a subset of the 24 3′UTR polymorphisms would be predicted to alter miRNA binding. To identify these SNPs, we entered both the NIH/Ola and SPRET/Outbred sequences into two miRNA binding prediction programs, MicroInspector [20] and MiRanda [19]. Fifteen polymorphisms were predicted to affect miRNA binding. *Cbll1* was the only gene with a 3′UTR variant fitting the linkage that did not have at least one candidate SNP predicted to disrupt or introduce a miRNA site (Table 2; Table S3; Figure S1).

**Table 2:** Candidate microRNAs for genes showing differential luciferase expression.

Gene	SNP	miRNA	Rationale for Candidacy	Predicted to Bind
*Bcap29*	1409C>T	***miR-128***	MicroSNiPer/MicroInspector^a^ RNA hybrid, miR expression^b^, cancer^c^	SPRET
	1409C>T	*miR-134*	MicroSNiPer/RNAcofold	SPRET
*Dgkb*	3170 T>G	***miR-489***	MicroInspector/RNAhybrid RNAcofold, cancer^c^	SPRET
	3170 T>G	miR-485*	MicroInspector^a^ miR expression^b^	SPRET
*Etv1*	3132 A/G	***miR-673-5p***	MicroSNiPer/RNAhybrid/RNAcofold	SPRET^d^
	3132 A/G	miR-674*	MicroSNiPer/RNAcofold	SPRET^d^
*Hbp1*	2437G>C	***miR-31***	MicroInspector, miR expression^b^, cancer^c^	NIH/Ola
	2437G>C	***miR-183***	MicroRNA, miR expression^b^, cancer^c^	NIH/Ola^d^
	2626 del7	*miR-5110*	MicroInspector/RNAcofold	NIH/Ola
	2626 del7	***miR-873***	Patrocles/RNAcofold, cancer^c^	NIH/Ola^d^
	2626 del7	miR-92b*	RNAhybrid, cancer^c^	NIH/Ola
	2626 del7	*miR-1224*	RNAhybrid, cancer^c^	NIH/Ola
*Ifrd1*	3239 G>T	***miR-875-3p***	MicroSNiPer/RNAhybrid/RNAcofold	SPRET^d^
	3261 A>G	*miR-3085-3p*	MicroSNiPer/RNAcofold	SPRET^d^
	3261 A>G	miR-664*	MicroSNiPer/RNAhybrid/RNAcofold	SPRET^d^
	3261 A>G	***miR-3064-5p***	MicroSNiPer/RNAhybrid/RNAcofold	SPRET^d^
*Pik3cg*	4978 C>T 4979 C>G	***miR-3064-3p***	MicroSNiPer/RNA hybrid/RNAcofold	SPRET
	4978 C>T 4979 C>G	***miR-707***	MicroInspector^a^	SPRET
*Tspan13*	958del2	***miR-1940***	MicroInspector^a^	NIH/Ola
*Twistnb*	1489 A>G	***miR-691***	Patrocles/RNA hybrid/RNAcofold	SPRET^d^
	1489 A>G	***miR-3074-5p***	MicroSNiPer/RNAhybrid/RNAcofold	SPRET^d^
	1489 A>G	***miR-718***	MicroInspector^a^	SPRET^d^

SPRET, SPRET/Outbred; miRNAs in bold are those that were evaluated, ^a^Free energy binding of target 3′UTR by MicroInspector was greater than −22 kcal/Joule; ^b^miR expression, The miRNA is expressed in skin; ^c^Cancer, The miRNA is differentially expressed in cancer, ^d^the SNP is predicted to be in the miRNA seed region.

**Figure 1:**
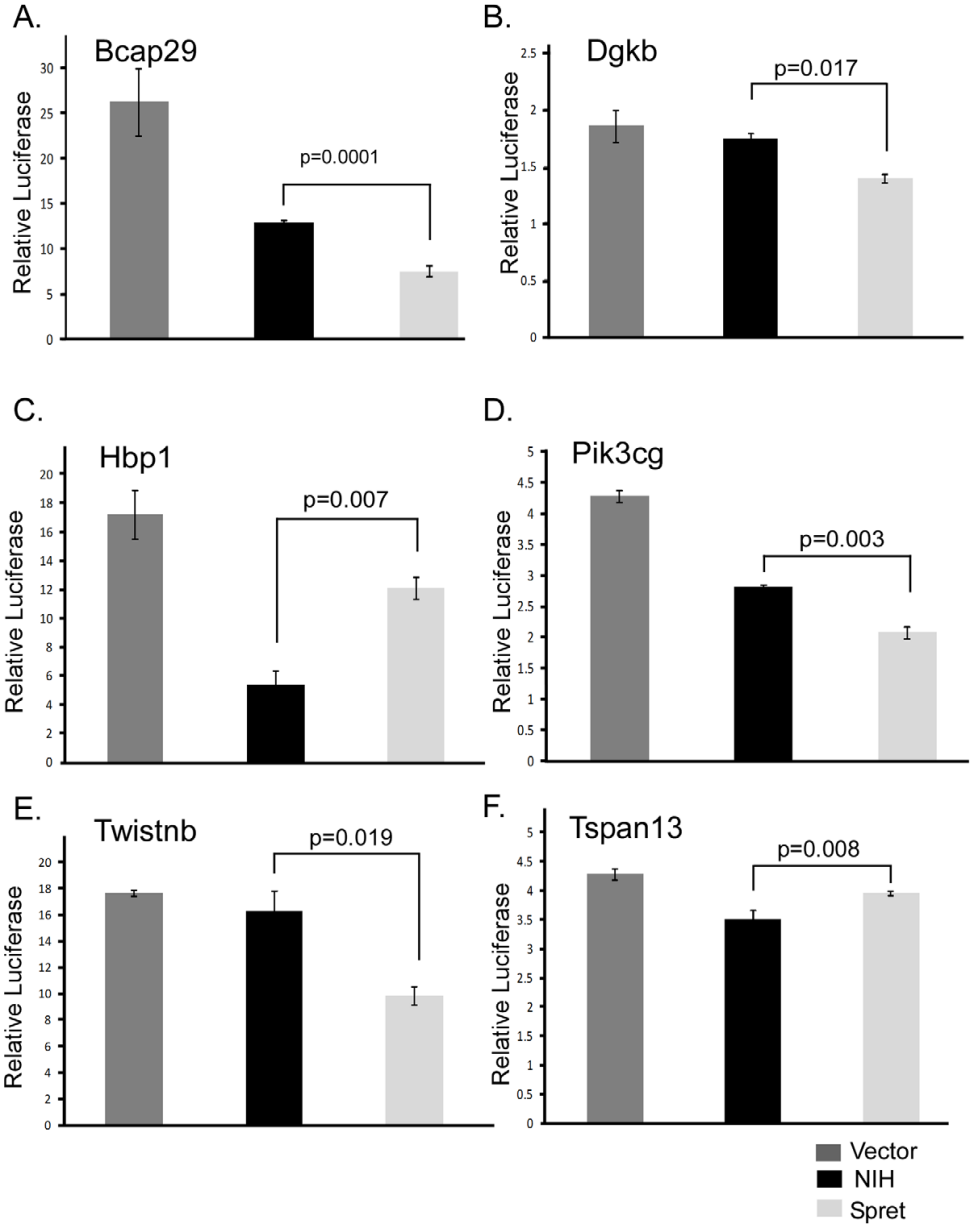
Luciferase assays for 3′UTRs of SPRET/Outbred and NIH/Ola. Representative relative luciferase units normalized to mock for the pGL3 luciferase vector (dark gray), NIH 3′UTR (Black) and SPRET/Outbred 3′UTRs (light gray) for six genes are shown. P-values of differential expression between NIH/Ola and SPRET/Outbred are indicated. A. *Bcap29*; B. *Dgkb*; C. *Hbp1*; D. *Pik3cg*; E. *Twistnb*; F. *Tspan13*.

To determine if 3′UTRs polymorphisms affected expression, we cloned fragments of the 3′UTRs of 245 to 1,652 bp in size which contained the variants of interest for the eight genes with polymorphisms fitting the linkage data and predicted to interfere with miRNA binding (Table S1). The 3′UTR for *Cbll1* was also cloned. Following cloning of 3′UTR fragments of *Bcap29*, *Cbll1*, *Dgkb*, *Hbp1*, *Meox2*, *Pik3cg*, *Stxbp1*, *Tspan13*, and *Twistnb*, into the pGL3-control luciferase vector, we transfected the constructs into C5N normal keratinocyte cells and measured luciferase levels of the two isoforms. We hypothesized that if the variant within the 3′UTR was predicted to show miRNA binding only in one strain, there should be less luciferase expression compared to the variant not predicted to bind the miRNA. The untranslated regions for *Bcap29*, *Dgkb*, *Hbp1, Pik3cg*, *Stxbp1*, and *Twistnb* had polymorphic sites which were predicted to both introduce and disrupt putative miRNA binding sites. Thus for these six genes, it was not clear how the polymorphism might affect expression. Of the nine 3′UTRs assessed, six showed statistically significant differential luciferase expression between the strains (Figure 1 and data not shown). The most pronounced differences in luciferase expression were in the 3′UTRs of *Hbp1* and *Bcap29* (Figure 1). For many of the genes, both 3′UTR isoforms showed decreased luciferase expression compared to the pGL3 vector suggesting that miRNAs or other regulatory mechanisms have effects on both 3′UTR forms.

### Identification of variants affecting putative microRNA binding sites

As variants in 3′UTR regions have been shown to affect miRNA binding and subsequent gene and protein expression [15], [16], we hypothesized that 3′UTR variants fitting the linkage data could affect gene regulation and therefore impact the difference in cancer susceptibility between NIH/Ola and SPRET/Outbred mice. Six of the nine 3′UTRs evaluated, *Bcap29*, *Dgkb*, *Hbp1*, *Pik3cg*, *Twistnb* and *Tspan1*, showed differential luciferase expression between NIH/Ola and SPRET/Outbred, yet as many of these had multiple SNPs that were predicted to affect binding, we wanted to prioritize which SNPs and miRNAs were most likely to do this. In addition, as some SNPs were previously determined to both introduce and disrupt potential binding sites, we wanted to use our luciferase data to choose miRNAs that would fit the expression data. To further refine our candidate SNP list and choose potential miRNAs for study, sequence from 3′UTRs of genes with candidate variants were assessed using additional miRNA binding prediction programs Patrocles, and MicroSNiPer. We also used MicroInspector, to identify binding sites with a MFE of less than −22 kcal/J in one form (strong binding) and greater than −18 kcal/J in the other form (weak or no binding), as recommended for Mus musculus [20]. For all predicted miRNA binding sites, we determined to what extent the polymorphism was predicted to affect the strength of binding. Using RNAhybrid and RNAcofold, we calculated a free energy of binding of the putative miRNAs. We tested expression differences of all miRNA interactions that showed predicted differences of greater than 5 kcal/J between the two variant forms in both prediction tools. All of the six genes with variants which fit the linkage data and showed differences in luciferase expression had variants with significant differences in predicted free energy binding of miRNAs, some of which contained SNPs in the predicted seed region (Table 2, Figure S1).

### Effect of microRNAs on expression

**Figure 2:**
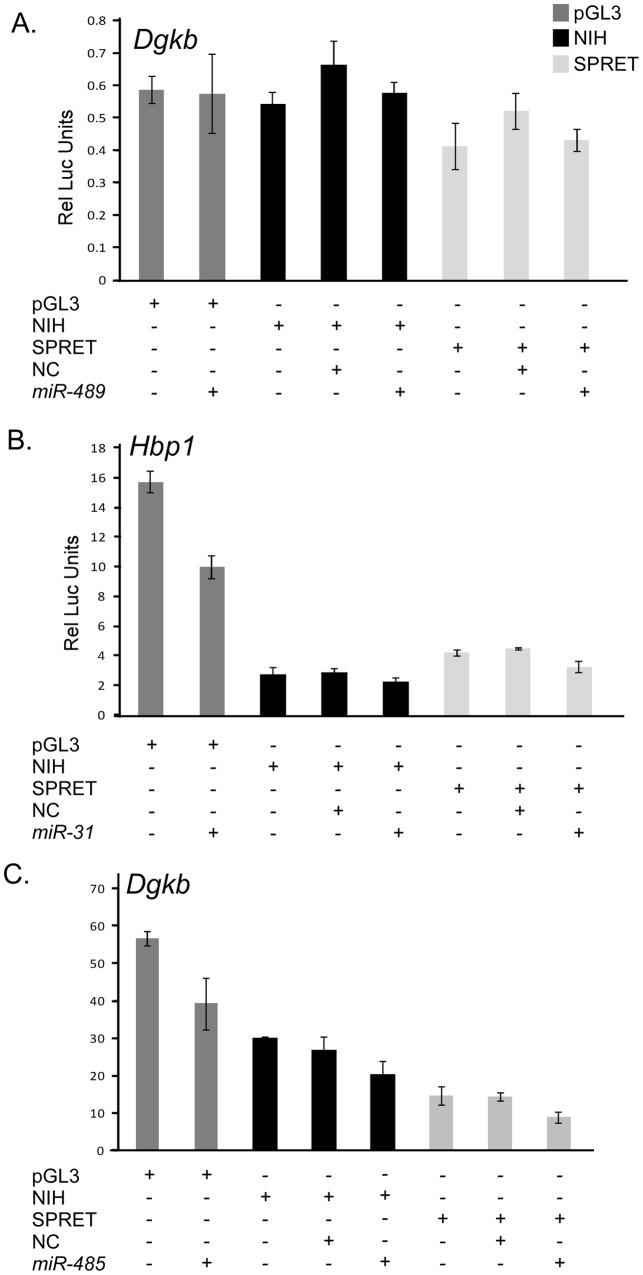
microRNA effect on luciferase expression. Representative experiments showing no effect of miRNA on luciferase expression and similar effects of the miRNA on both isoforms are shown. A. *Dgkb* 3′UTR with *miR-489*; B. *Tspan13* 3′UTR with *miR-1940; C. Dgkb with miR-485*. pGL3, pGL3 luciferase vector without insert; NIH, NIH/Ola 3′UTR; SPRET, SPRET/Outbred 3′UTR; NC, scrambled control miRNA; Dark Gray bars, pGL3 luciferase vector; Black bars, pGL3 vector with the NIH/Ola 3′UTR; Light gray bars, pGL3 vector containing the SPRET/Outbred 3′UTR.

To determine if the predicted miRNAs could influence luciferase expression differences of *Bcap29*, *Dgkb*, *Hbp1*, *Pik3cg*, *Twistnb* and *Tspan13*, we co-transfected C5N cells with the NIH/Ola or SPRET/Outbred luciferase constructs and a precursor miRNA predicted to bind to the variants consistent with linkage and our initial luciferase expression data (Table 2). We chose 13 candidate miRNAs for evaluation. We began our studies by only evaluating the isoform predicted to be bound by the miRNA and evaluated both isoforms when we saw an effect in the expected direction of the miRNA on luciferase levels. miRNAs for *Pik3cg* (*miR-707*), *Hbp1* (*miR-127*, *miR-183*, and *miR-873*), *Twistnb* (*miR-718*, and *miR-691*), and *Dgkb* (*miR-489*) had no impact on luciferase expression (Figure 2A and data not shown). *miR-1940* with the *Tspan13* 3′UTR and *miR-31* with the *Hbp1* 3′UTR decreased luciferase expression of both isoforms similarly suggesting that these miRNAs bound the 3′UTRs to the same degree (Figure 2B and data not shown). Other miRNAs, *miR-128 (Bcap29), miR-3064-3p (Pik3cg)*, *miR-3074-5p* (*Twistnb*) and *miR-485 (Dgkb)* decreased the luciferase expression of the pGL3 control empty vector to the same degree as the vector containing the cloned 3′UTR (Figure 2C and data not shown). These data suggest that the miRNAs we chose for analysis were not responsible for the observed differences in luciferase expression between SPRET/Outbred and NIH/Ola.

**Figure 3:**
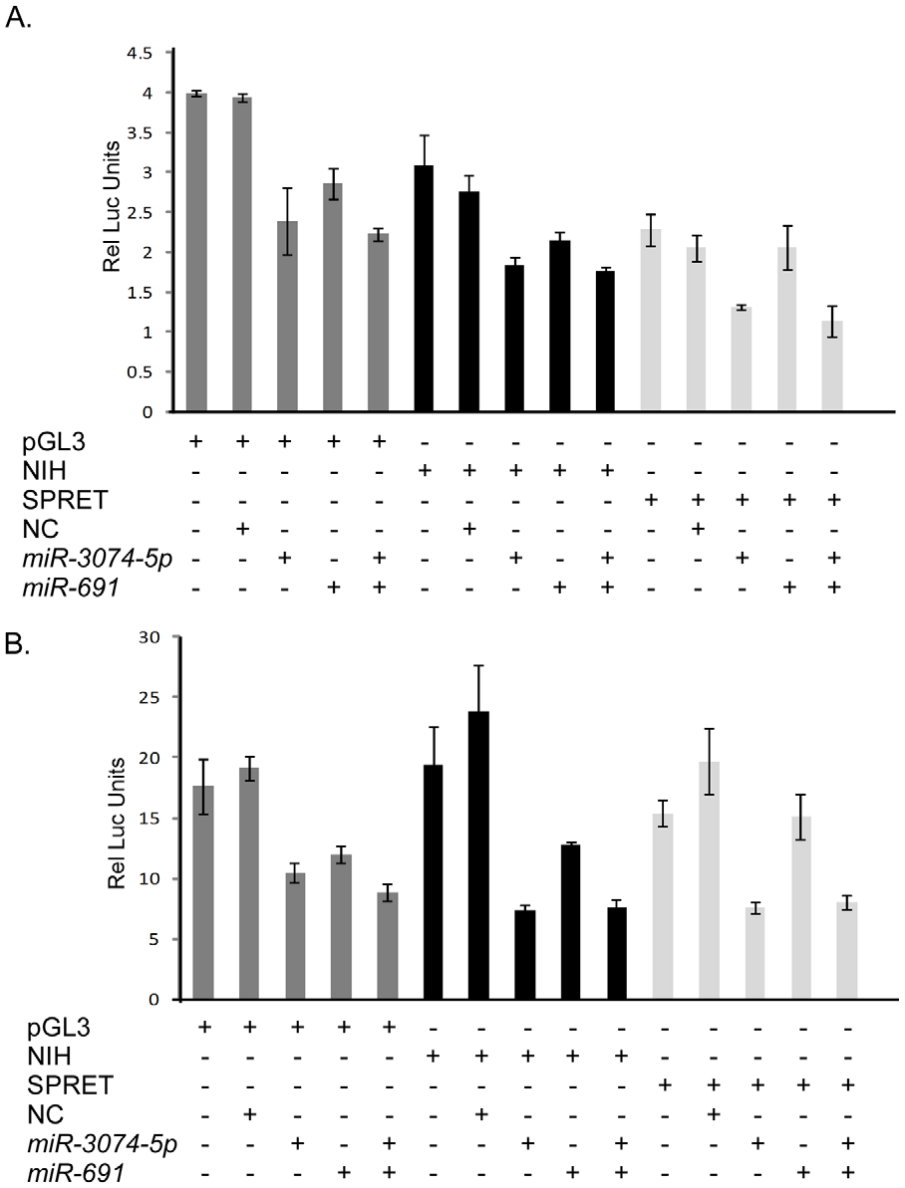
*Twistnb* expression under differential experimental conditions. Representative experiments showing non-specific effect of *miR-3074-5p* and/or *miR-691* on both isoforms of *Twistnb* are shown at a low dose transfection of miRNA precursors. A. *Twistnb* 3′UTRs at 24 hours post-transfection with *miR-3074-5p*, *miR-691* or both miRNAs. B. *Twistnb* 3′UTRs at 48 hours post-transfection with *miR-3074-5p*, *miR-691* or both miRNAs. pGL3, pGL3 luciferase vector without insert; NIH, NIH/Ola 3′UTR; SPRET, SPRET/Outbred 3′UTR; NC, scrambled control miRNA; Dark Gray bars, pGL3 luciferase vector; Black bars, pGL3 vector with the NIH/Ola 3′UTR; Light gray bars, pGL3 vector containing the SPRET/Outbred 3′UTR.

To rule out the possibility that we were missing differential effects of the miRNAs on luciferase expression because of the experimental conditions used, we evaluated the *Twistnb* 3′UTR using additional doses of miRNAs *miR-3074-5p* and *miR-691* and additional time-points post transfection. This 3′UTR was chosen for more detailed study because it contained variants predicted to bind to the seed region of both of these miRNA (Figure S1). We observed no differences in the effect of the miRNAs compared to our original experiments (Figure 3A, 3B and data not shown). As miRNAs may act in combination, we also evaluated *miR-3074-5p* and *miR-691* in combination on the *Twistnb* 3′UTR NIH/Ola and SPRET/Outbred isoforms and found that the results for the combination of *miR-691* and *miR-3074-5p* were identical to those of the *miR-3074-5p* alone (Figure 3A, 3B and data not shown). These results suggest that our data is not likely to be an artifact of the experimental conditions used.

To further evaluate the effect of *miR-3074-5p* on pGL3 and NIH/Ola and SPRET/Outbred *Twistnb* 3′UTRs, we transfected an inhibitor for *miR-3074-5p* and compared expression to a negative control inhibitor and *miR-3074-5p*. If the miRNA were directly targeting the predicted SPRET/Outbred *Twistnb* isoform and the observed effect on the pGL3 vector and the NIH/Ola *Twistnb* were non-specific effects (Figure 3A and B), one would expect that addition of the inhibitor would have the greatest effect on the pGL3 vector with the SPRET/Outbred 3′UTR. Addition of the anti-*miR-3074-5p* showed the greatest fold increase in luciferase expression of the pGL3 vector and showed non-specific increases in expression of the SPRET/Outbred and NIH/Ola isoforms suggesting that the reduced luciferase expression is non-specific (data not shown).

It is possible that endogenous miRNAs may be exerting maximal knock-down and addition of miRNA precursor to our C5N cells would not induce further decreases in luciferase expression. To evaluate this possibility we measured miRNA expression of our RNA harvested from C5N cells that were mock-transfected. Of note, all of the miRNAs evaluated showed evidence of expression in the non-transfected by qPCR, but most of these were expressed at relative levels of 1% or less of the control, *sno-202*. Thus, for the majority of the miRNAs in this study, endogenous levels of expression in the C5N cells are unlikely to causing maximal knockdown of the predicted target 3′UTR. MicroRNAs showing higher level of endogenous expression included *miR-31* (252% of control), *miR-183* (12.5% of control), *miR-675-3p* (2.2% of control) and *miR-3074-5p* (3.7% of control).

### mRNA expression of candidate target genes

**Figure 4:**
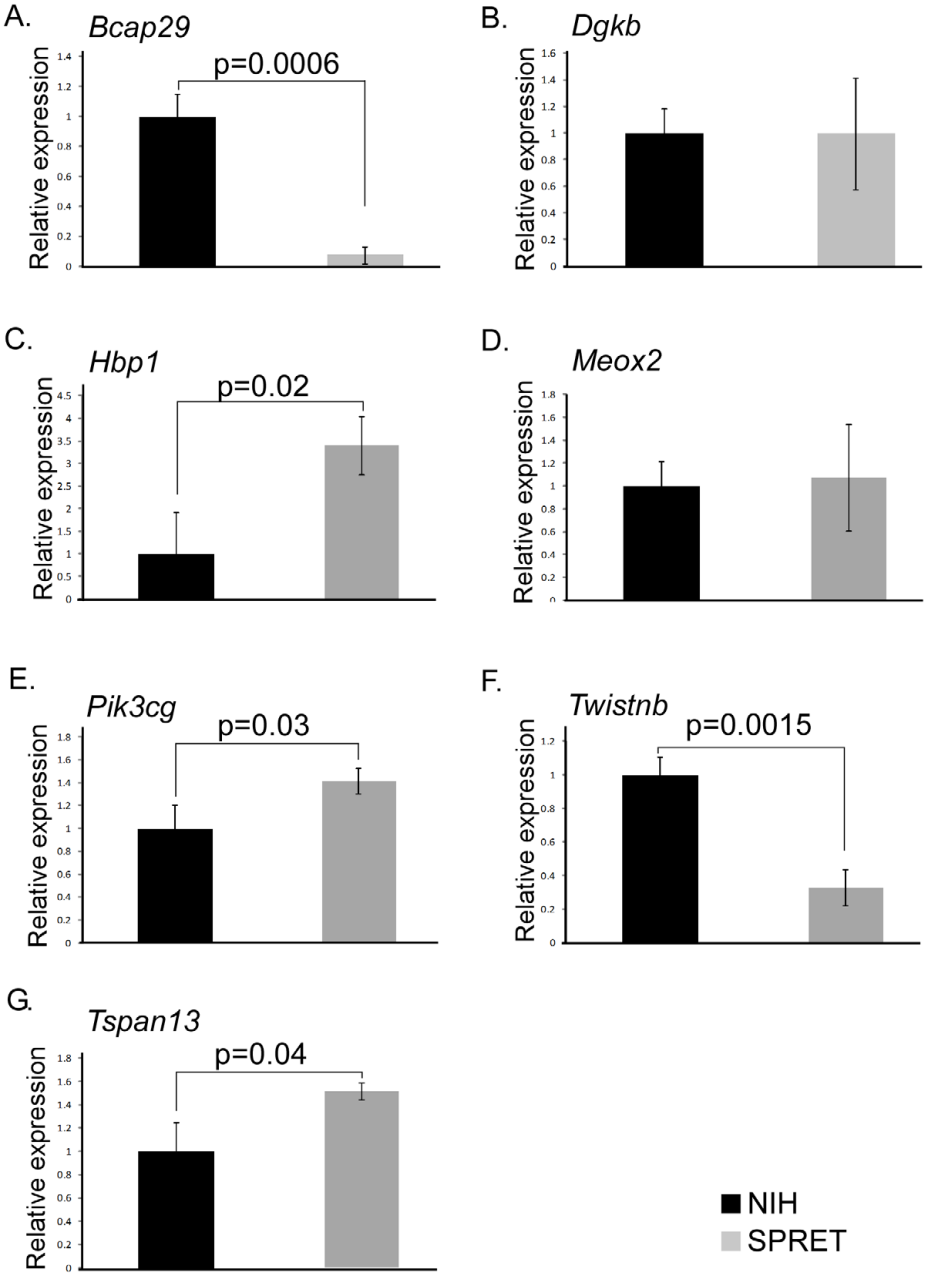
mRNA expression of candidate genes with luciferase expression differences. Quantitative PCR of seven genes evaluated for differential luciferase expression between SPRET/Outbred and NIH/Ola 3′UTR are shown. *Hprt*, *Ppia* and *L19* were used as loading controls; results normalizing to *Hprt* are shown. Percentage relative expression of *Hprt* for NIH/Ola and Spret/Outbred was normalized to NIH/Ola expression. P-values are indicated. A. *Bcap29*, B. *Dgkb*, C. *Hbp1*, D. *Meox2*, E. *Pik3cg*, F. *Tspan13*, G. *Twistnb*. Black bars, NIH/Ola; Gray bars, SPRET/Outbred.

One effect of miRNA binding to mRNA is degradation of the mRNA product and decreased expression. Genes mapping to *Skts5* were assessed by qPCR to determine if there were differences in tail mRNA between NIH/Ola and SPRET/Outbred. Of the tested genes containing candidate 3′UTR variants we found significant differences in mRNA expression in *Bcap29*, *Hbp1*, *Pik3cg*, *Twistnb and Tspan13*, but no significant differences in expression of *Dgkb* and *Meox2* (Figure 4 and data not shown). Expression for *Stxbp6* was too low for comparison. Genes that showed consistent results between the luciferase expression assays and the qPCR are *Bcap29* (higher expression in NIH/Ola), *Hbp1* (higher expression in SPRET/Outbred), *Meox2* (no significant difference in expression), *Twistnb* (higher expression in NIH/Ola) and *Tspan13* (higher expression in SPRET/Outbred). *Bcap29* showed the highest degree of difference, approximately 15-fold higher expression in NIH/Ola than SPRET/Outbred (Figure 4). *Dgkb* showed no significant difference in mRNA expression, but higher luciferase expression of the NIH 3′UTR. *Pik3cg* showed higher expression of SPRET/Outbred mRNA, but lower luciferase expression. Thus, five of the seven genes assessed by qPCR showed consistent expression patterns between mRNA and the effect of the 3′UTR on luciferase expression. As miRNAs work in two manners, one by increasing mRNA degradation and the other by interfering with translation, it is possible that no difference in mRNA expression would be observed even when differential miRNA binding takes place [26], [27].

### Identification of additional candidate genes showing mRNA expression differences

**Figure 5:**
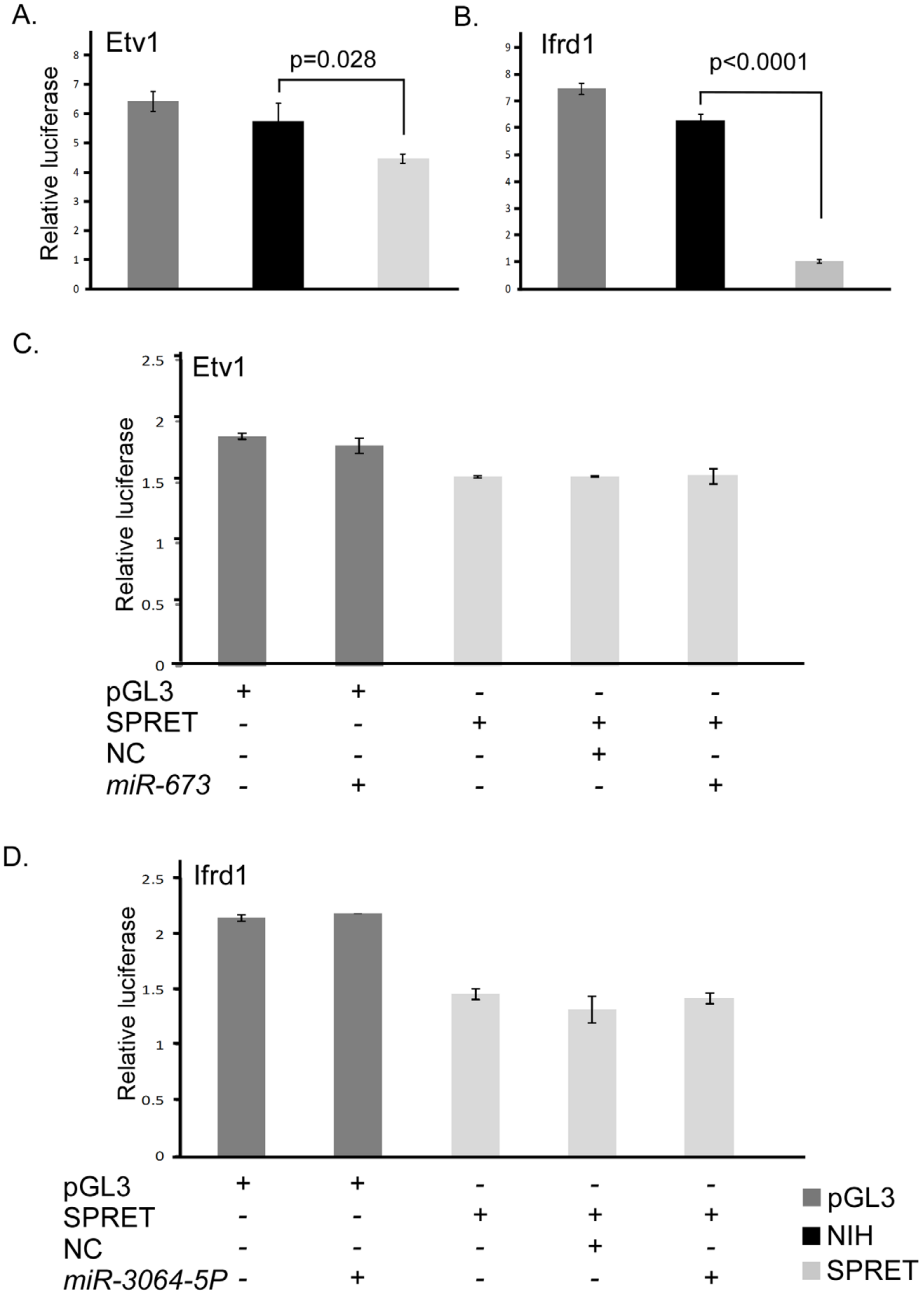
Luciferase and mRNA results of *Etv1* and *Ifrd1*. Representative relative luciferase units normalized to mock for the pGL3 luciferase vector (dark gray), NIH 3′UTR (Black) and SPRET/Outbred 3′UTRs (light gray) for A. Etv1 and B. Ifrd1 are shown. Representative experiments showing no effect of miRNA on luciferase expression for the predicted SPRET/Outbred target for C. *Etv1* and *miR-673* and D. *Ifrd1* with *miR-3064-5P*. ApGL3, pGL3 luciferase vector without insert; NIH, NIH/Ola 3′UTR; SPRET, SPRET/Outbred 3′UTR; NC, scrambled control miRNA, Dark Gray bars, pGL3 luciferase vector; Black bars, pGL3 vector with the NIH/Ola 3′UTR; Light gray bars, pGL3 vector containing the SPRET/Outbred 3′UTR.

The SPRET/EiJ by FVB/N F1 backcrosses did not show evidence of linkage at *Skts5*. In contrast, the SPRET/EiJ by NIH/Ola linkage analysis was equivocal for this region. Thus, we cannot completely rule out the possibility that SPRET/EiJ may share a resistance allele with SPRET/Outbred and that the difference in linkage results is due to differences in susceptibility of the susceptible strains NIH/Ola and FVB/N at this locus. To assess the much larger list of potential candidate genes at Skts5 that would be consistent with SPRET/EiJ and SPRET/Outbred sharing a resistance allele, we evaluated the 3′UTRs for genes that had not been assessed earlier. There were 17 additional genes that had polymorphisms that were shared in SPRET/Outbred and SPRET/EiJ but were not present in STF/PAS, so we prioritized genes for evaluation if they also showed mRNA expression differences between SPRET/Outbred and NIH/Ola by qPCR or if they had previously been shown to be involved in cancer. We identified four genes, *EG629820*, *Etv1*, *Ifrd1*, and *Pbef1*, that fit these criteria and which contained polymorphisms in SPRET/Outbred and SPRET/EiJ but not in STF/PAS. We cloned the 3′UTRs for these genes and evaluated their effect on luciferase expression. We found differential luciferase expression in *Etv1* and *Ifrd1* (Figure 5 and data not shown). We next evaluated the SNPs in *Ifrd1* and *Etv1* for their predicted effect on miRNA binding using MicroInspector, Patrocles, and microSNiPer and identified 11 SNPs that were predicted to differentially bind to a total of 43 miRNAs (Table 2; Table S3). Using RNAhybrid and RNAcofold, we found two miRNAs in *Ifrd1* (*miR-3064-5p* and *miR-875-3p*) and one in *Etv1* (*miR-673-5p*) with predicted differences of greater than 5 kcal/joule MFE between the two mouse strains in both tools. We evaluated these microRNAs for their effect on luciferase expression and found no effects on expression of the predicted target strain (Figure 5 and data not shown).

## Discussion

We assessed the role of 24 variants found only in SPRET/Outbred mapping to nine genes located at locus *Skts5* for their effect on expression. We found significant differences in luciferase expression for six of the 3′UTRs of these genes, but we did not find binding of predicted miRNAs which accounted for these differences. An anti-miR for *miR-3074-5p* resulted in non-significant increases in both SPRET/Outbred and NIH/Ola *Twistnb* isoforms and had the greatest effect on the pGL3 vector providing additional evidence that the decreased expression of the SPRET/Outbred *Twistnb* isoform is not likely due to this miRNA. We further evaluated 3′UTRs from four additional genes that contained polymorphisms observed in SPRET/Outbred and SPRET/EiJ but not in STF/PAS and identified two 3′UTRs, *Etv1* and *Ifrd1* that showed differential lucifierase expression between SPRET and NIH/Ola but exhibited no differences in expression with predicted miRNAs. These results indicate that variants in 3′UTRs can affect expression *in vitro* and that observed differential mRNA expression of *Hbp1, Tspan13, Pik3cg, Bcap29* and *Twistnb* between NIH/Ola and SPRET/Outbred and of *Etv1* and *Ifrd1* between NIH/Ola and SPRET/Outbred-SPRET/EiJ may be due to variants in the 3′UTR.

*Skts5* shows evidence of an epistatic genetic interaction with a second locus, *Skts1*, on mouse chromosome 7. Thus, it is possible that all the *spretus* strains will share the causal susceptibility polymorphism at *Skts5* and only the strains that have the resistance allele at *Skts1* will show evidence of linkage at *Skts5* [4], [25]. The linkage at *Skts5* may depend on genetic interactions at other susceptibility loci and the reason we did not observe linkage for the NIH/Ola by STF/PAS cross was that the STF/PAS mice do not have the other important locus. In this case, all 3′UTR polymorphisms between SPRET/Outbred and NIH/Ola could be considered as candidates variants for *Skts5*. The approach taken in this study would have missed this type of causal variant. All polymorphisms that differed between NIH/Ola and SPRET/Outbred, including those that were shared between all *spretus* strains were included in our 3′UTR for all genes but *EG629820* and *Etv1* which had SNPs changed by site directed mutagenesis because we made our PCR products for cloning from genomic DNA. These sites may be responsible for the differences in luciferase expression observed in this study rather than the site that was unique to SPRET/Outbred mouse.

SNPs in the 3′UTR region may impact gene expression through other mechanisms than disruption of miRNA binding. SNPs in the prothrombin 3′UTR affect post-transcriptional processing and 3′-cleavage/polyadenylation and SNPs in *SNCA* affect polyadenylation [28], [29]; both of which affect expression. Variants in the 5′UTR of the *COMT* gene are associated with structural destabilization of the *COMT* mRNA through differential tertiary structures [30]. Thus, it is possible that the SNPs evaluated in this study which correlated with differential luciferase expression may affect mRNA stability through polyadenylation or other differences resulting in changes to mRNA stability which can be addressed in future studies.

Variants in the 3′UTR of *Dgkb* affected both luciferase expression and predicted binding of miR-485* and *miR-489*. However, *Dgkb* was one of the genes that did not show a corresponding difference in mRNA expression as measured by qPCR. As many miRNAs do not affect mRNA levels, but exert their effects on translation and protein levels it is possible that miRNAs not evaluated in this study could bind to *Dgkb* mRNA and result only in impaired translation and differential protein levels [26].

There are some limitations to this study. In our study, we evaluated the 3′UTRs from 13 genes, nine fitting conservative linkage requirements and four fitting relaxed criteria. Six of the genes in our study are reported in the Ensembl database to have additional isoforms with shorter or different 3′UTRs that do not include the SNPs fitting the linkage (Table S1). For these six genes (*Cbll1*, *Etv1*, *Hbp1*, *Ifrd1*, *Pik3cg*, *Stxbp6*) it is possible that the miRNAs and SNPs being studied for these genes are not biologically relevant. Importantly, this would effectively rule these variants out as candidates for *Skts5*. Differences in luciferase expression may be caused by a combination of miRNAs acting synergistically on the 3′UTR. With the exception of *Twistnb* with *miR-3074-5p* and *miR-691*, we only tested miRNAs individually. There was no synergistic effect seen for these miRNA combinations on *Twistnb*, but we cannot rule out the possibility of synergistic effects for the other 3′UTRs. For miRNAs in which we did not observe a decreased expression in luciferase, it is possible that the suppression of expression was already at maximal levels and that addition of the mature miRNA could not enhance this suppression. We feel that the possibility of this is low, as many of the miRNAs were expressed endogenously in C5N at extremely low levels and some were not detectable. Furthermore, in our study we evaluated seven miRNAs for their effect on only the predicted target 3′UTR. These seven miRNA did not show any effect on the luciferase expression of the predicted target 3′UTR and were not evaluated further. It is possible that these miRNAs could target a non-predicted binding site including the SNP which would be an interesting but surprising result.

As new miRNAs continue to be mapped and as miRNA-binding prediction algorithms are constantly being refined as we understand more about the biology of miRNA binding to targets, many of the polymorphisms mapping to 3′UTRs for which we did not identify any putative miRNA-binding sites may be target sites which we missed. However, only eight of the polymorphisms in 3′UTRs which we assessed failed to have any predicted binding sites so the majority of those fitting the linkage data were evaluated in our assays. We evaluated the effect of the miRNAs on luciferase expression at 24 hours post-transfection. miRNAs may take longer to exert an effect on expression so we could have missed these effects. To test the effects of different time points and miRNA concentrations we evaluated two miRNAs, m*iR-3074-5p* and *miR-691*, at multiple time points and concentrations and observed no differences in effect on 3′UTRs for the differential experimental conditions. Thus, it is less likely that experimental conditions are influencing our findings. It is also possible that the criteria for inclusion of miRNAs for study were too stringent. In the majority of the 3′UTRs which were cloned there are additional polymorphic sites between *Mus musculus* and *Mus spretus* that did not fit with the original mouse linkage. Thus, variants observed in all of the spretus strains may be contributing to the differences in observed luciferase expression.

Another area highlighted by this study is the need for *in silico* prediction programs with higher sensitivity and specificity. We used a fairly stringent approach that utilized hits from multiple programs to identify candidate SNPs affecting miRNA binding. As none of the miRNA/SNP pairs we tested were validated using our experimental design, this is a fairly high false positive rate. With the increased utilization of next-generation exome and genome sequencing, the number of variants in 3′UTRs that may affect gene regulation is likely to increase and robust tools to identify these are critical.

In summary, 3′UTR variants present between skin cancer susceptible NIH/Ola and skin cancer resistant SPRET/Outbred mice may result in the observed differences in mRNA expression between these strains, but the mechanism for this is unknown. These variants are also potential candidates for the observed difference in SCC susceptibility. Some of the variants map to putative miRNA binding sites and are predicted to disrupt binding; however, none of the individual miRNAs tested in this study appeared to be driving the observed differences in expression. The reason for the differences in expression by the different 3′UTRs is unknown and warrants future study. 3′UTR variants fitting the linkage data may contribute to differences in expression for genes including *Bcap29, Dgkb, Hbp1, Pik3cg, Twistnb, and Tspan13*, and, as such, remain candidates for the observed skin cancer susceptibility locus at *Skts5*. *Etv1* and *Ifrd1* remain potential candidates when considering potential epigenetic interactions or differences between susceptibility strains NIH/Ola and FVB/N at this locus [25]. Although this study did not uncover any 3′UTR variants disrupting miRNA binding, we did identify a large number of 3′UTR variants affecting luciferase expression. Thus, future studies evaluating 3′UTRs variants as functional candidates should consider multiple mechanisms for effects on mRNA and translation.

## Supporting Information

Figure S1 **miRNA/mRNA alignments by RNAhybrid.** Predicted mRNA/miRNA alignments by RNA hybrid are illustrated in text (left panels and pictorial (right panesl) representations. The yellow highlighted base represents the polymorphism between NIH/Ola and SPRET/Outbred. Green represents the miRNA and red the mRNA of each binding pair. Mfe, mean free energy of binding.
(PDF)

Table S1 **3′UTR regions cloned.**
(DOCX)

Table S2 **Primers for 3′UTR cloning.**
(DOCX)

Table S3 **Mean Free Energy Differences.**
(DOCX)
