# Supplementary material for: The Impact of 3′UTR Variants on Differential Expression of Candidate Cancer Susceptibility Genes
Source: PLoS One. 2013 Mar 5;8(3):e58609. doi: 10.1371/journal.pone.0058609 (PMC3589377; doi:10.1371/journal.pone.0058609)

Figure S1: RNAhybrid mRNA/miRNA alignments

Bcap29

Bcap29: SPRET with miRNA-134

```
position  431
target 5' A  A                 U 3'
           CC      CAAUUAGUCAC
           GG      GUUGGUCAGUG
miRNA  3' G  GAGACCA           U 5'


mfe: -20.2 kcal/mol
```

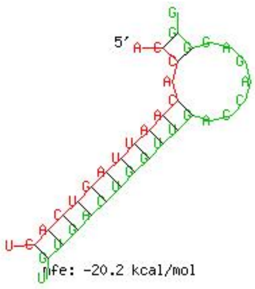

Bcap29: NIH with miRNA-134 No Predicted Alignment

Bcap29: SPRET with miR-128

```
position  425
target 5'   U   UA   ACAAUUAG     C   3'
             GAG  ACC        UCACUG
             CUC  UGG        AGUGAC
miRNA  3' UUU        CCA          ACU 5'


mfe: -16.5 kcal/mol
```

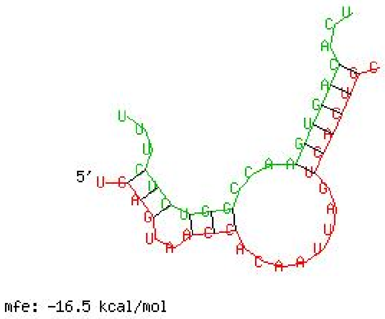

Bcap29: NIH with miR-128- no predicted alignment

Dgkb

Dgkb SPRET with *miR-489*

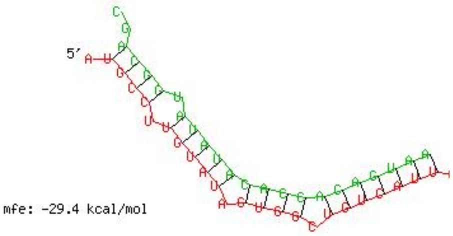


```
position  96
target 5'  A    U     A    C       G 3'
            UGCC UGUAU GUGG UGUCAUU
            ACGG AUAUA CACC ACAGUAA
miRNA  3'   CG    U
```

mfe: -29.4 kcal/mol

Dgkb NIH with *miR-489*

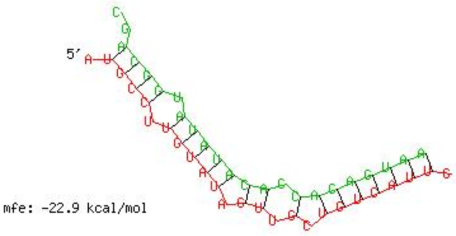


```
position  96
target 5'  A    U     A  U C       G 3'
            UGCC UGUAU GU G UGUCAUU
            ACGG AUAUA CA C ACAGUAA
miRNA  3' CG    U        C           5'
```

mfe: -22.9 kcal/mol

Etv1

Etv1 SPRET with *miR-673-5p*

```
position  43
target 5'  A         UU       C  3'
            CC    ACC   GCUGUG
            GG    UGG   CGACAC
miRNA  3' GA  UUCC   UCU      UC 5'
mfe: -18.5 kcal/mol
```

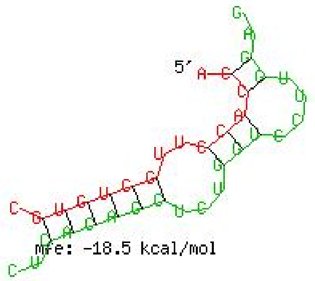

Etv1 NIH with *miR-673-5p*

```
position  43
target 5'  A   CCUU    U    A     3'
            CCA    GCUA  GCU
            GGU    UGGU  CGA
miRNA  3' GA   UCC     CU   CACUC 5'


mfe: -13.1 kcal/mol
```

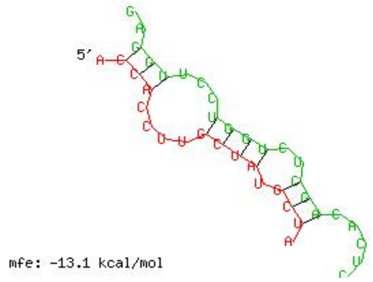

Etv1 SPRET with *miR-674*

```
position  41
target 5'  U          G    U    U 3'
            UACCAC CUU    C GUGC
            GUGGUG GGG    G CACG
miRNA  3' AU      A   UAGA U      5'


mfe: -20.6 kcal/mol
```

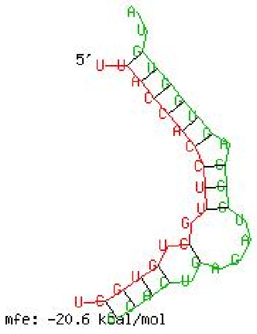

Etv1 NIH with *miR-674*

```
position  44
target 5'  C    UU   AUG    AAG   CUUCUA   A 3'
            CACC  GCU   CUAU   CAG      UGC
            GUGG  UGA   GGUA   GUC      ACG
miRNA  3' AU         G      GA               5'

mfe: -19.0 kcal/mol
```

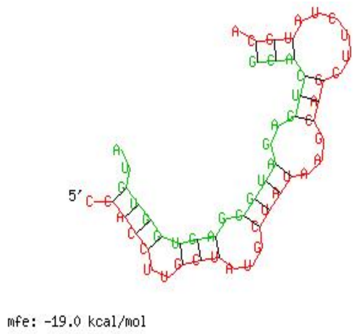

Hbp1

Hbp1 SPRET with *miR-31*

```
position  517
target 5' A   GGAUG     C     A      C 3'
           GGU     GUGUC GUGUC UUGUCU
           UCG     UACGG CGUAG AACGGA
miRNA  3' G   A         U              5'
mfe: -23.7 kcal/mol
```

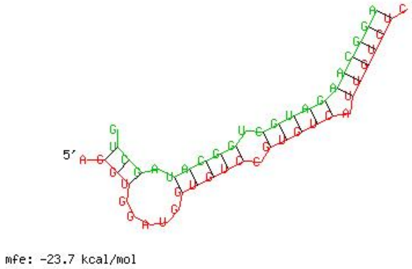

Hbp1 NIH with *miR-31*

```
position  517
target 5' A   GGAUG           A      C 3'
           GGU     GUGUCGGUGUC UUGUCU
           UCG     UACGGUCGUAG AACGGA
miRNA  3' G   A

mfe: -27.7 kcal/mol
```

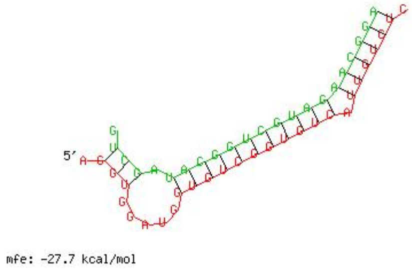

Hbp1 SPRET with *miR-183*

```
position  517
target 5' A       GG  U          U 3'
           GGUGGAU  UG CC GUGUCAU
           UCACUUA  AU GG CACGGUA
miRNA  3'         AG     U       U 5'


mfe: -22.9 kcal/mol
```

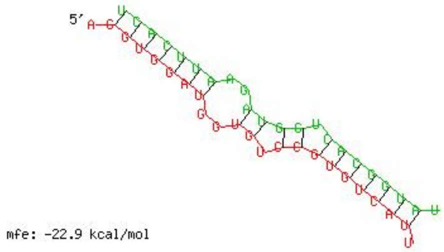

Hbp1 NIH with *miR-183*

```
position  517
target 5' A       GG            U 3'
           GGUGGAU  UGUCGGUGUCAU
           UCACUUA  AUGGUCACGGUA
miRNA  3'         AG            U 5'


mfe: -26.0 kcal/mol
```

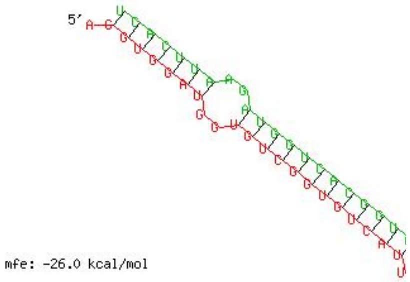

Hbp1 SPRET with *miR-5110*

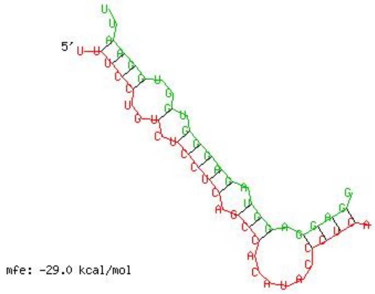


```
position  718
target 5'  U    UG C     A   ACAUAC    A 3'
            UUCC  U UCCUC GCC      CCUC
            AAGG  G GGGAG UGG      GGAG
miRNA  3' UU    UG U     A   A         G 5'


mfe: -29.0 kcal/mol
```

Hbp1 NIH with *miR-5110*

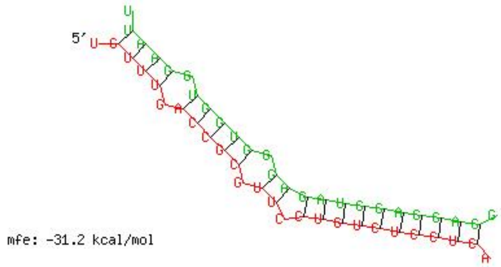


```
position  714
target 5' U    G     G  C          A 3'
           GUUU ACCGC UU CUGUCUCCUC
           UAAG UGGUG GA GAUGGAGGAG
miRNA  3' U    G     G             G 5'


mfe: -31.2 kcal/mol
```

Hbp1 SPRET with *miR-873* -no predicted alignments

Hbp1 NIH with *miR-873*

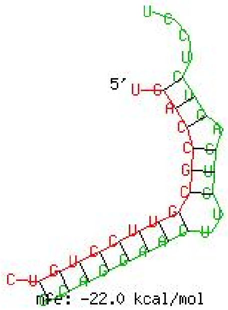


```
position  718
target 5'    U                 C 3'
              GAC CGC  GUUCCUGU
              CUG GUG  CAAGGACG
miRNA  3' UCCU   A   UU          5'
mfe: -22.0 kcal/mol
```

Hbp1 **SPRET with *miR-92b***

```
position  690
target 5'   U    U     GU  ACACCAUUGUGUUUU       U   C 3'
             GCUG UACCA  CG                UCCUGUC CCU
             UGAC GUGGU  GC                AGGGCAG GGA
miRNA  3' UUG

mfe: -28.0 kcal/mol
```

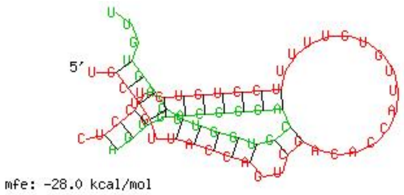

Hbp1 **NIH with *miR-92b***

```
position  709
target 5' C  UG   UUG      U       U   C 3'
           AU  UGU   ACCGCG UCCUGUC CCU
           UG  ACG   UGGUGC AGGGCAG GGA
miRNA  3' U  UG

mfe: -35.1 kcal/mol
```

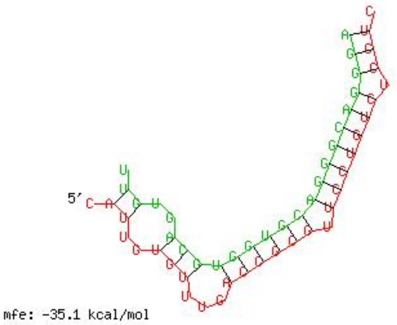

Hbp1 **SPRET with *miR-1224***

```
position  719
target 5' U   UGU        CCACAUAC      C 3'
           UCC   CUCCUCAG        CCUCAU
           AGG   GAGGGGUC        GGAGUG
miRNA  3' G   UG         A               5'
mfe: -27.8 kcal/mol
```

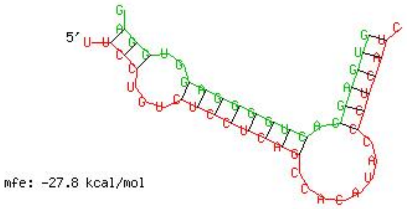

Hbp1 **NIH with *miR-1224***

```
position  720
target 5'  A    G     GUC      G 3'
            CCGC UUCCU   UCCUCA
            GGUG AGGGG   AGGAGU
miRNA  3' GA    G     UC       G 5'
mfe: -27.8 kcal/mol
```

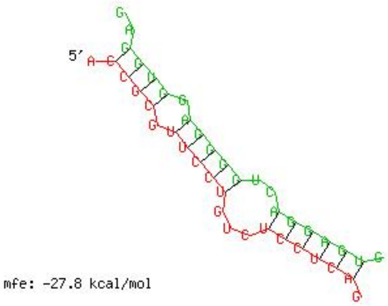

Ifrd1

Ifrd1 SPRET with *miR-875-3p*

```
position  181
target 5'     C      AUUG       A  3'
               CUUCGG    AUUUUCA
               GGAGUC    UAAAAGU
miRNA  3' GUAUC      A          CC 5'

mfe: -16.2 kcal/mol
```

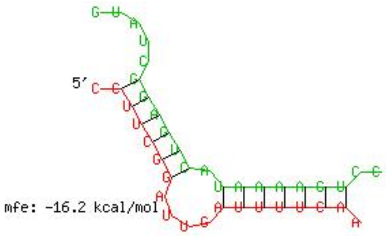

Ifrd1 NIH with *miR-875-3p*-no predicted binding

Ifrd1 SPRET with *miR-3085-3p*

```
position  199
target 5'      A    GUUCACAC        G 3'
                GUCA        UAGCCAGA
                CGGU        GUCGGUCU
miRNA  3' CUCCCC    AUC               5'


mfe: -19.9 kcal/mol
```

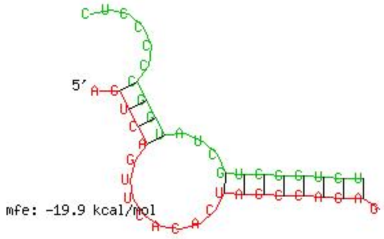

Ifrd1 NIH with *miR-3085-3p*

```
position  200
target 5'      A    G   UCACACUAA     G 3'
                GUCA AGU         CCAGA
                CGGU UCG         GGUCU
miRNA  3' CUCCCC    A   UC              5'


mfe: -16.0 kcal/mol
```

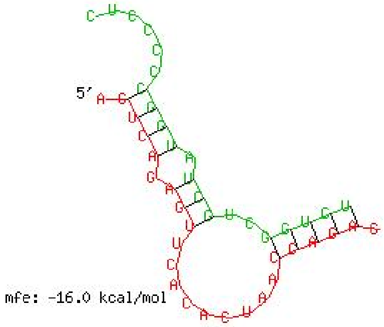

Ifrd1 SPRET with *miR-664**

```
position  198
target 5'  A     G    ACA        A 3'
            AGUCA  UUC   CUAGCCAG
            UCAGU  AAG   GGUCGGUC
miRNA  3' GG     AA   G            5'
mfe: -27.0 kcal/mol
```

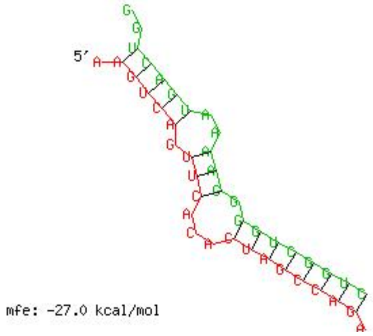

Ifrd1 NIH with *miR-664**

```
position  217
target 5' A    AG           G    3'
           CCAG     UUUCUUAG
           GGUC     AAGGGGUC
miRNA  3'      AGUAA        GGUC 5'
mfe: -16.2 kcal/mol
```

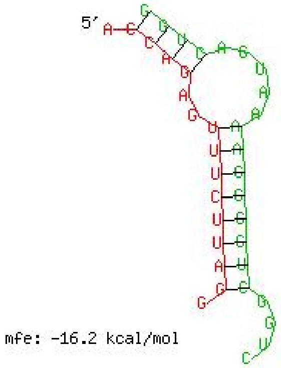

Ifrd1 SPRET with *miR-3064-5p*

```
position  199
target 5'   A U  GU     C        G 3'
             G CA  UCACA UAGCCAGA
             C GU  GGUGU GUCGGUCU
miRNA  3' AAA    GU     U          5'


mfe: -27.2 kcal/mol
```

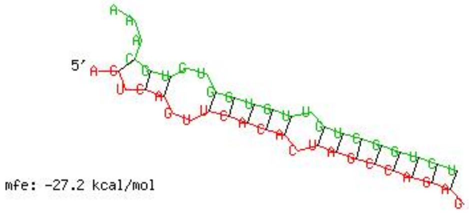

Ifrd1 NIH with *miR-3064-5p*

```
position  207
target 5' G          A          G 3'
           UU CACACUA      CCAGA
           AA GUGUGGU      GGUCU
miRNA  3' A  C       GUUGUC       5'

mfe: -19.6 kcal/mol
```

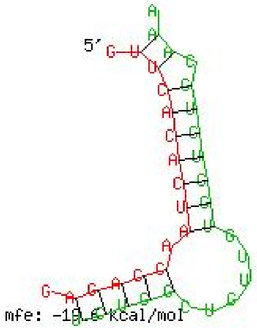

Twistnb

Twistnb SPRET with *miR-691*

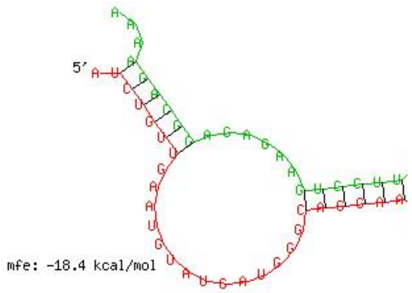


```
position  323
target 5'   A      GAAUGUAUGAUGGG      A 3'
             UCUGUU              CAGGAA
             AGACGG              GUCCUU
miRNA  3' AAA      AGAGAA              A 5'

mfe: -18.4 kcal/mol
```

Twistnb NIH with *miR-691* no predicted binding

Twistnb SPRET with *miR-3074-5p*

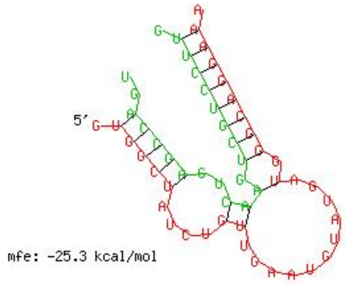


```
position  317
target 5'  G     AUCU  UGAAUGUAUGA G        A 3'
            UGGCU    GU            U GGCAGGAA
            ACCGA    CA            A UCGUCCUU
miRNA  3' UG     GU                 G        G 5'
mfe: -25.3 kcal/mol
```

Twistnb NIH with *miR-3074-5p*

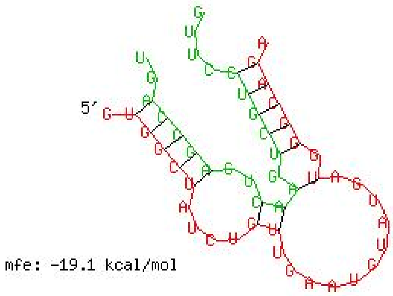


```
position  317
target 5'  G     AUCU  UGAAUGUAUGA G     A    3'
            UGGCU    GU            U GGCAG
            ACCGA    CA            A UCGUC
miRNA  3' UG     GU                 G     CUUG 5'


mfe: -19.1 kcal/mol
```

Twistnb SPRET with *miR-718*

```
position  316
target 5' U UG   AUCU  UGAAUGUAUGA     A    A 3'
           G  GCU    GU           UGGGC GGAA
           C  UGG    CG           GCCCG CCUU
miRNA  3' G UG   GC                         C 5'


mfe: -22.6 kcal/mol
```

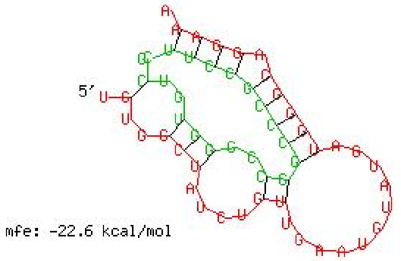

Twistnb NIH with *miR-718*

```
position  316
target 5' U UG   AUCU  UGAAUGUAUGA     A   A 3'
           G  GCU    GU           UGGGC GAA
           C  UGG    CG           GCCCG CUU
miRNA  3' G UG   GC                    C   C 5'


mfe: -21.1 kcal/mol
```

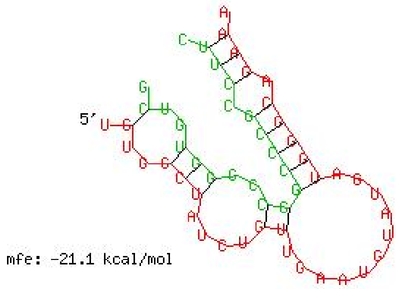

Supplement: Figure S1 — miRNA/mRNA alignments by RNAhybrid. Predicted mRNA/miRNA alignments by RNA hybrid are illustrated in text (left panels and pictorial (right panesl) representations. The yellow highlighted base represents the polymorphism between NIH/Ola and SPRET/Outbred. Green represents the miRNA and red the mRNA of each binding pair. Mfe, mean free energy of binding. (PDF) [file pone.0058609.s001.pdf]
