# Supplementary material for: The Impact of 3′UTR Variants on Differential Expression of Candidate Cancer Susceptibility Genes
Source: PLoS One. 2013 Mar 5;8(3):e58609. doi: 10.1371/journal.pone.0058609 (PMC3589377; doi:10.1371/journal.pone.0058609)
Supplement: Table S1 — 3′UTR regions cloned. (DOCX) [file pone.0058609.s002.docx]

Table S1: Details on 3’UTR Cloning

| Gene | Ensembl transcript (version) | Region cloned  (bp position in cDNA) | Size of  region  cloned | 3’UTR bp position in cDNA | # of other protein coding isoforms^d^, |
| --- | --- | --- | --- | --- | --- |
| *Bcap29* | ENSMUST00000020979 (201) | 1049-2164^a^ | 1115 | 967-1954 | 1  SNPs in both isoforms |
| *Cbll1* | ENSMUST00000101499 (203) | 1668-2494 | 826 | 1558-4007 | 2, SNPs in 2 isoforms |
| *Dgkb* | ENSMUST00000040500 (201) | 3039-3283 | 244 | 2825-5544 | None |
| *Etv1(1)^b^* | ENSMUST00000095767 (001) | 2826-4230 | 1404 | 1862-4307 | Yes, 7; SNPs in two isoforms |
| *Etv1 (2)^b^* | ENSMUST00000095767 (001) | 3444-4230 | 786 | 1862-4307 | Yes, 7; SNPs in two isoforms |
| *Gm7008 (EG629820)* | ENSMUST00000038121 (001) | 991-1176 | 185 | 784-1366 | None |
| *Hbp1* | ENSMUST00000172314 (001) | 1979-2715 | 736 | 1907-3142 | 4; SNPs in one isoform |
| *Ifrd1* | ENSMUST00000001672 (001) | 2413-3729^a^ | 1316 | 1584-3378 | 1; SNPs in one isoform |
| *Meox2* | ENSMUST00000041183 (001) | 1687-2004 | 317 | 1204-4611 | None |
| *Nampt (Pbef1)* | ENSMUST00000020886 (201) | 1670-2344^c^ | 674 | 1711-4547 | None |
| *Pikc3g* | ENSMUST00000053215 (001) | 4851-5560 | 709 | 3463-5871 | Yes, 1; SNPs in one isoform |
| *Stxbp6* | ENSMUST00000120531 (002) | 847-2498 | 1651 | 844-4209 | Yes; 2; SNPs in one isoform |
| *Tspan13* | ENSMUST00000020896 (001) | 774-1349 ^c^ | 575 | 793-1932 | None |
| *Twistnb* | ENSMUST00000020877 (201) | 1454-1703 | 249 | 1025-2324 | None |

^a^cloned region extends past 3’end of transcript, ^b^Etv1 3’UTR was cloned in two fragments, ^c^cloned region begins prior to 3’UTR, ^d^Only isoforms in which the 3’UTRs have been mapped are listed
