# Supplementary material for: The Impact of 3′UTR Variants on Differential Expression of Candidate Cancer Susceptibility Genes
Source: PLoS One. 2013 Mar 5;8(3):e58609. doi: 10.1371/journal.pone.0058609 (PMC3589377; doi:10.1371/journal.pone.0058609)
Supplement: Table S2 — Primers for 3′UTR cloning. (DOCX) [file pone.0058609.s003.docx]

Table S2: Cloning Primers

| Gene | Forward Primer (5’-3’) | Reverse Primer (5’-3’) |
| --- | --- | --- |
| *Bcap29* | tgtaatactagtccgCCGCACAGGCTATACAGTAGTGACA | gtctgctcgaagcggTGTCAGCCCACAAGGTGTAACT |
| *Cbll1* | tgtaatactagtccgGCAGATAAGTGGCTTGGTTGAGCA | gtctgctcgaagcggTGACTACAAGGAACACCGTCTGCT |
| *Dgkb* | tgtaatactagtccgTGCATGCACTCCCATAGGGTTT | gtctgctcgaagcggTTTCTTAAGCCACGGGTCGAACAC |
| *Etv1-1* | tgtaatactagtccgGCTGTTCTAGTTACAGCAAGCACTC | gtctgctcgaagcggGCTAGCTGACGATTTACAGAGTCCA |
| *Etv1-2* | tgtaatactagtccgAGCGTGTGCCTTGCTGTTTCATTC | gtctgctcgaagcggGCTAGCTGACGATTTACAGAGTCCA |
| *Gm7008 (EG629820)* | tgtaatactagtccgAGGGTTCTCAGTAGGTGAGGCAAA | gtctgctcgaagcggAGAACACACCCATCGCAAACTCTG |
| *Hbp1* | tgtaatactagtccgGGAAGACCTTGGTCTCACCATTTGT | gtctgctcgaagcggGGTGCACTGCAGTAAGTGCAAAGT |
| *Ifrd1* | tgtaatactagtccgTGCATTTCAGGGTACAGGGACTCA | gtctgctcgaagcggTGCCTGGACTTGCGGGTATATGAA |
| *Meox2* | tgtaatactagtccgCCACAGTGCCTGAAATCACCAAGT | gtctgctcgaagcggTTTGCCGCACAAGACTCGTTCT |
| *Nampt (Pbef1)* | tgtaatactagtccgAATGCACAGCTGAACATCGAGCAG | gtctgctcgaagcggTTCGGAACTCTGTTGGGTGTCTGT |
| *Pikc3g* | tgtaatactagtccgTGTGTCTCCAGAAGCCAAGTAGCA | gtctgctcgaagcggACTCACAGGAATGGCAAGGTAGCA |
| *Stxbp6* | tgtaatactagtccgACTGCCTATCCTGGTGAAGCTCTA | gtctgctcgaagcggGCATGTGTAGCTTGCCTCATTGCT |
| *Tspan13* | tgtaatactagtccgCCGAGTGCTTTCCTTTGACGAGAA | gtctgctcgaagcggACAGGGATGCAATCCTGACAGACT |
| *Twistnb* | tgtaatactagtccgACCCTGTGGCTATCTGTTGAATGT | gtctgctcgaagcggGCTCCTCCTCAAAGCAAGTCTAGT |
