# Supplementary material for: The Impact of 3′UTR Variants on Differential Expression of Candidate Cancer Susceptibility Genes
Source: PLoS One. 2013 Mar 5;8(3):e58609. doi: 10.1371/journal.pone.0058609 (PMC3589377; doi:10.1371/journal.pone.0058609)
Supplement: Table S3 — Mean Free Energy Differences. (DOCX) [file pone.0058609.s004.docx]

**Table S3: Mean Free Energy Differences**

|  |  | RNAcofold | |  | RNA hybrid | |  |
| --- | --- | --- | --- | --- | --- | --- | --- |
| **MicroRNA** | **SNP** | **MFE NIH** | **MFE SPRET** | **∆MFE** | **MFE NIH** | **MFE SPRET** | **∆MFE** |
| **Bcap29** |  |  |  |  |  |  |  |
| MicroSNiPer |  |  |  |  |  |  |  |
| mmu-miR-134 | 1409 C>T | -11.5 | -16.5 | 5 | -16 | -20.2 | 4.2 |
| mmu-miR-27b | 1409 C>T | -16.9 | -18.1 | 1.2 | -21.5 | -22.7 | 1.2 |
| mmu-miR-27a | 1409 C>T | -16.9 | -18.1 | 1.2 | -21.5 | -22.7 | 1.2 |
| mmu-miR-128 | 1409 C>T | -6.7 | -12.3 | 5.6 | -11 | -17.9 | 6.9 |
| **Dgkb** |  |  |  |  |  |  |  |
| MicroInspector |  |  |  |  |  |  |  |
| mmu-miR-489 | 3170 T>G | -18.17 | -24.1 | 5.93 | -22.2 | -28.7 | 6.5 |
| **Etv1** |  |  |  |  |  |  |  |
| MicroSNiPer |  |  |  |  |  |  |  |
| mmu-miR-363-5p | 2927 A>T | -13.8 | -13.8 | 0 | -18.3 | -18.3 | 0 |
| mmu-miR-449b | 2927 A>T | -12.3 | -13.5 | 1.2 | -17.6 | -18.8 | 1.2 |
| mmu-miR-338-5p | 2927 A>T | -6 | -6.8 | 0.8 | -10.7 | -12.1 | 1.4 |
| mmu-miR-673-5p | 3132 A>G | -7.7 | -13.7 | 6 | -12.6 | -18.5 | 5.9 |
| mmu-miR-21 | 3132 A>G | -7.5 | -8.2 | 0.7 | -11.8 | -13.5 | 1.7 |
| mmu-miR-29c | 3132 A>G | -5.8 | -8.5 | 2.7 | -10.2 | -12.8 | 2.6 |
| mmu-miR-29a | 3132 A>G | -6.5 | -9.2 | 2.7 | -10.2 | -12.8 | 2.6 |
| mmu-miR-674* | 3132 A>G | -3.9 | -9 | 5.1 | -8.3 | -13.1 | 4.8 |
| mmu-miR-1192 | 3407 C>T | -8.3 | -12.4 | 4.1 | -13.7 | -17.5 | 3.8 |
| mmu-miR-384-5p | 3407 C>T | -7.9 | -9.3 | 1.4 | -12.5 | -14.4 | 1.9 |
| mmu-miR-1969 | 3428 G>T | -11.2 | -11.6 | 0.4 | -16.3 | -17.2 | 0.9 |
| Patrocles |  |  |  |  |  |  |  |
| [mmu-miR-495](http://microrna.sanger.ac.uk/cgi-bin/sequences/mirna_entry.pl?acc=MIMAT0003456) | 3407 C>T | -8.3 | -10.1 | 1.8 | -13 | -14.4 | 1.4 |
| [mmu-miR-1192](http://microrna.sanger.ac.uk/cgi-bin/sequences/mirna_entry.pl?acc=MIMAT0005850) | 3407 C>T | -8.3 | -12.4 | 4.1 | -13.7 | -17.5 | 3.8 |
| **Hbp1** |  |  |  |  |  |  |  |
| MicroInspector |  |  |  |  |  |  |  |
| mmu-miR-31 | 2437 G>C | -21.7 | -17.8 | 3.9 | -27.6 | -23.6 | 4 |
| mmu-miR-714 | 2437 G>C | -14.2 | -17.8 | 3.6 | -20.3 | -22.6 | 2.3 |
| mmu-miR-449c | 2437 G>C | -17.91 | -18.5 | 0.59 | -22.5 | -20.9 | 1.6 |
| mmu-miR-146b | 2437 G>C | -17.91 | -16.3 | 1.61 | -23.2 | -19.4 | 3.8 |
| mmu-miR-762 | 2437 G>C | -22.7 | -25.3 | 2.6 | -27.3 | -27.3 | 0 |
| mmu-miR-341 | 2626 del7 | -17.82 | -12.92 | 4.9 | -22.5 | -18.7 | 3.8 |
| mmu-miR-92b* | 2626 del7 | -29.1 | -25.7 | 3.4 | -34.7 | -25.2 | 9.5 |
| mmu-miR-326* | 2626 del7 | -23.3 | -24.5 | 1.2 | -25.2 | -25 | 0.2 |
| mmu-miR-504* | 2626 del7 | -19.8 | -21.7 | 1.9 | -24.7 | -24.2 | 0.5 |
| mmu-miR-5110 | 2626 del7 | -27.6 | -20.3 | 7.3 | -31.2 | -26.5 | 4.7 |
| mmu-miR-1224 | 2626 del7 | -23 | -23.4 | 0.4 | -27.8 | -22.6 | 5.2 |
| mmu-miR-1934* | 2626 del7 | -24 | -24 | 0 | -28.8 | -28.8 | 0 |
| MicroSNiPer |  |  |  |  |  |  |  |
| mmu-miR-489 | 2437 G>C | -19.1 | -16.4 | 2.7 | -24.3 | 20.8 | 3.5 |
| mmu-miR-667 | 2437 G>C | -20.3 | -19.4 | 0.9 | -28.1 | -27.2 | 0.9 |
| mmu-miR-878-3p | 2437 G>C | -18.5 | -16.7 | 1.8 | -23.6 | -22.3 | 1.3 |
| mmu-miR-883a-5p | 2437 G>C | -15.4 | -15.5 | 0.1 | -20.2 | -20.2 | 0 |
| Patrocles |  |  |  |  |  |  |  |
| [mmu-miR-667](http://microrna.sanger.ac.uk/cgi-bin/sequences/mirna_entry.pl?acc=MIMAT0003734) | 2437 G>C | -24.7 | -23.8 | 0.9 | -15.5 | -16.1 | 0.6 |
| [mmu-miR-873](http://microrna.sanger.ac.uk/cgi-bin/sequences/mirna_entry.pl?acc=MIMAT0004936) | 2626 del7 | -18.4 | -12.9 | 5.5 | -21.9 | -18.2 | 3.7 |
| **Ifrd1** |  |  |  |  |  |  |  |
| MicroSNiPer |  |  |  |  |  |  |  |
| mmu-miR-487b* | 2625 C>A | -6 | -10.4 | 4.4 | -10.7 | -14.7 | 4 |
| mmu-miR-34a | 2625 C>A | -15 | -18.6 | 3.6 | -20.6 | -24.3 | 3.7 |
| mmu-miR-449a | 2625 C>A | -19.7 | -17.6 | 2.1 | -24.4 | -23 | 1.4 |
| mmu-miR-146a* | 3025 T>A | -13.1 | -12.7 | 0.4 | -17.8 | -17.4 | 0.4 |
| mmu-miR-1897-3p | 3025 T>A | -15.6 | -15.6 | 0 | -21.4 | -21.4 | 0 |
| mmu-miR-3098-3p | 3025 T>A | -11.6 | -11.6 | 0 | -13.4 | -14.6 | 1.2 |
| mmu-miR-694 | 3239 G>T | -5.54 | -10 | 4.46 | -10.1 | -14.7 | 4.6 |
| mmu-miR-875-3p | 3239 G>T | -4.9 | -11 | 6.1 | -10.8 | -16.2 | 5.4 |
| mmu-miR-488 | 3239 G>T | -5.4 | -6.9 | 1.5 | -10.9 | -11.6 | 0.7 |
| mmu-miR-664* | 3250, 3261 del2, A>G | -16 | -20.9 | 4.9 | -21.2 | -25.9 | 4.7 |
| mmu-miR-3085-3p | 3261 A>G | -7.6 | -12.2 | 4.6 | -12.2 | -17.3 | 5.1 |
| mmu-miR-3064-5p | 3261 A>G | -13.2 | -18.8 | 5.6 | -18.8 | -26.2 | 7.4 |
| mmu-miR-149 | 3261 A>G | -12.4 | -14.2 | 1.8 | -17.6 | -19.6 | 2 |
| mmu-miR-5102 | 3261 A>G | -9 | -11.9 | 2.9 | -13.7 | -17.6 | 3.9 |
| mmu-miR-206* | 3293 G>A | -6.9 | -7.3 | 0.4 | -13 | -13.4 | 0.4 |
| mmu-miR-499 | 3293 G>A | -15.2 | -15.6 | 0.4 | -21.5 | -21.9 | 0.4 |
| mmu-miR-208b | 3293 G>A | -10.3 | -8 | 2.3 | -13.3 | -13.7 | 0.4 |
| mmu-miR-702 | 3374 G>A | -9.9 | -9.9 | 0 | -14.5 | -14.9 | 0.4 |
| mmu-miR-495* | 3374, 3376 G>A, T>C | -17.3 | -19.9 | 2.6 | -21.7 | -24.3 | 2.6 |
| mmu-miR-543* | 3374, 3376 G>A, T>C | -15.9 | -18.3 | 2.4 | -21 | -23.6 | 2.6 |
| mmu-miR-496* | 3374, 3376 G>A, T>C | -17.6 | -20.3 | 2.7 | -23.4 | -26.1 | 2.7 |
| mmu-miR-804 | 3374, 3376 G>A, T>C | -10.9 | -13.5 | 2.6 | -15.5 | -18.1 | 2.6 |
| mmu-miR-453 | 3374, 3376 G>A, T>C | -10.7 | -13.4 | 2.7 | -16.3 | -19 | 2.7 |
| mmu-miR-188-3p | 3374, 3376 G>A, T>C | -13.4 | -15.4 | 2 | -17.9 | -19.7 | 1.8 |
| mmu-miR-744* | 3374, 3376 G>A, T>C | -8.2 | -10.7 | 2.5 | -12.8 | -14.8 | 2 |
| mmu-miR-3089-5p | 3374, 3376 G>A, T>C | -8.4 | -8.4 | 0 | -10.3 | -12.7 | 2.4 |
| mmu-miR-1195 | 3374, 3376 G>A, T>C | -11.9 | -13.8 | 1.9 | -13.6 | -15.1 | 1.5 |
| Patrocles |  |  |  |  |  |  |  |
| [mmu-miR-687](http://microrna.sanger.ac.uk/cgi-bin/sequences/mirna_entry.pl?acc=MIMAT0003466) | 2625 C>A | -11.9 | -14.2 | 2.3 | -13.6 | -16 | 2.4 |
| [mmu-miR-694](http://microrna.sanger.ac.uk/cgi-bin/sequences/mirna_entry.pl?acc=MIMAT0003474) | 3239 G>T | -5.84 | -10.3 | 4.46 | -10.4 | -15 | 4.6 |
| [mmu-miR-149](http://microrna.sanger.ac.uk/cgi-bin/sequences/mirna_entry.pl?acc=MIMAT0000159) | 3261 A>G | -15 | -19.1 | 4.1 | -18.4 | -20.4 | 2 |
| **Pik3cg** |  |  |  |  |  |  |  |
| MicroSNiPer |  |  |  |  |  |  |  |
| mmu-miR-3064-3p | 4978, 4979 C>T, C>G | -9.2 | -15.4 | 6.2 | -13.8 | -21.8 | 8 |
| **Tspan13** |  |  |  |  |  |  |  |
| MicroSNiPer |  |  |  |  |  |  |  |
| mmu-miR-692 | 1207 A>C | -8.5 | -5.7 | 2.8 | -13.1 | -14.9 | 1.8 |
| **Twistnb** |  |  |  |  |  |  |  |
| MicroInspector |  |  |  |  |  |  |  |
| mmu-miR-718 | 1489 A>G | -16.7 | -19 | 2.3 | -21 | -22.5 | 1.5 |
| mmu-miR-1943 | 1489 A>G | -15.1 | -15.8 | 0.7 | -18.6 | -22.3 | 3.7 |
| MicroSNiPer |  |  |  |  |  |  |  |
| mmu-miR-3074-5p | 1489 A>G | -8.1 | -13.8 | 5.7 | -13.8 | -20 | 6.2 |
| Patrocles |  |  |  |  |  |  |  |
| [mmu-miR-691](http://microrna.sanger.ac.uk/cgi-bin/sequences/mirna_entry.pl?acc=MIMAT0003470) | 1489 A>G | -7.7 | -13.1 | 5.4 | -13.2 | -18.4 | 5.2 |
| [mmu-miR-145*](http://microrna.sanger.ac.uk/cgi-bin/sequences/mirna_entry.pl?acc=MIMAT0004534) | 1489 A>G | -11.2 | -11.6 | 0.4 | -16.3 | -16.3 | 0 |

MFE, mean free energy; Yellow indicates **∆**MFE of greater than 5. The program to identify the microRNA predicted to bind to the each SNP (MicroSNiPer, MicroInspector, Patrocles) is listed under the genes and the program used to generate the MFE predicted is listed across the top (RNAcofold; RNA hybrid).
